# Withdrawal of life-support in paediatric intensive care - a study of time intervals between discussion, decision and death

**DOI:** 10.1186/1471-2431-11-39

**Published:** 2011-05-21

**Authors:** Felix Oberender, James Tibballs

**Affiliations:** 1 Intensive Care Unit, Royal Children's Hospital, Melbourne, Victoria, VIC 3052, Australia; 2 Departments of Paediatrics & Pharmacology, University of Melbourne Royal Children's Hospital, Melbourne, Victoria, VIC 3052, Australia

**Keywords:** withdrawal and withholding of life-sustaining treatment, time, end-of-life care, terminal care, death, organ donation after cardiac death

## Abstract

**Background:**

Scant information exists about the time-course of events during withdrawal of life-sustaining treatment. We investigated the time required for end-of-life decisions, subsequent withdrawal of life-sustaining treatment and the time to death.

**Methods:**

Prospective, observational study in the ICU of a tertiary paediatric hospital.

**Results:**

Data on 38 cases of withdrawal of life-sustaining treatment were recorded over a 12-month period (75% of PICU deaths). The time from the first discussion between medical staff and parents of the subject of withdrawal of life-sustaining treatment to parents and medical staff making the decision varied widely from immediate to 457 hours (19 days) with a median time of 67.8 hours (2.8 days). Large variations were subsequently also observed from the time of decision to actual commencement of the process ranging from 30 minutes to 47.3 hrs (2 days) with a median requirement of 4.7 hours. Death was apparent to staff at a median time of 10 minutes following withdrawal of life support varying from immediate to a maximum of 6.4 hours. Twenty-one per cent of children died more than 1 hour after withdrawal of treatment. Medical confirmation of death occurred at 0 to 35 minutes thereafter with the physician having left the bedside during withdrawal in 18 cases (48%) to attend other patients or to allow privacy for the family.

**Conclusions:**

Wide case-by-case variation in timeframes occurs at every step of the process of withdrawal of life-sustaining treatment until death. This knowledge may facilitate medical management, clinical leadership, guidance of parents and inform organ procurement after cardiac death.

## Background

Withdrawal of life-sustaining treatment has become the predominant end-of-life scenario in children's hospitals in the developed world. A variety of studies over the last two decades have highlighted the intensive care setting as the central, and in some instances nearly exclusive, place for making life and death decisions within a modern children's hospital [1-5]. Although physician, nursing, ethical and legal aspects have since been important foci of research in this difficult-to-study area [6,7], scant information exists about the time-course of the process of withdrawal of life-sustaining treatment from the moment of discussion to actual death. Limited knowledge of this aspect of care contributes to the formidable challenges of medical decision-making, bedside management, the provision of clinical leadership and guidance for parents at critical and painful moments. In addition, the advent and promotion of organ procurement after cardiac death sees the specialty engaged in a controversy about its role in the management of the dying process [8-10] and this also warrants a deeper understanding of the time-course of the events leading to death after withdrawal of life-sustaining treatment.

In this paper, we present the results of a 12-month prospective, observational study aiming to elucidate the time required for end-of-life decisions and subsequent withdrawal of life-sustaining treatment in an Australian paediatric intensive care unit.

## Methods

A prospective, observational study of deaths occurring in the Paediatric Intensive Care Unit (PICU) was conducted at the Royal Children's Hospital (RCH) Melbourne, Australia. The RCH is a 250-bed tertiary teaching hospital, which serves a population of approximately 6 million. The PICU is an 18-bed comprehensive intensive care facility admitting approximately 1400 children per year. It is engaged in the full spectrum of paediatric critical care including cardiac and trauma care.

Information was obtained for deaths occurring during a period of 12 months (2007). Data collection for each case commenced when the decision to withdraw life-sustaining treatment had been taken by medical staff and parents. While retrospective information about the discussions regarding withdrawal of life-sustaining treatment had to be obtained from the medical record, all other data from that point on was collected contemporaneously. This involved one of the two researchers being either present at the time of withdrawal of life-sustaining treatment or collecting the data immediately afterwards, i.e. at the beginning of the following shift in case the researcher was not present when death occurred. Data collected included diagnostic category, age at the time of death, time of first discussion between medical staff and parents about withdrawal of life-support, time of the decision made by medical staff and parents to proceed with withdrawal, the initiation of withdrawal followed by the times of apparent and confirmed death. The term apparent death denotes the appearance of death to bedside personnel (physician or nurse) before confirmation by clinical examination. In addition, information was collected about presence of monitoring and staff during withdrawal of life-sustaining treatment. The data analysis excluded cases of withholding of life-sustaining treatment as varying levels and modes of life-support may continue to be provided to patients in this category in our unit. In contrast, withdrawal of life-support in our unit invariably entailed the discontinuation of all life-support (ventilation, inotropic infusions, extracorporeal life-support).

Numerical data was assumed to be non-parametric with calculation of median values and statement of minimum and maximum values. Data regarding the reasons for withdrawal of life-support, pharmacological management as well as number, occupation and seniority of staff present at the bedside was also obtained but is the subject of a separate paper. The research was approved by the RCH Ethics in Human Research Committee and written informed consent was not required for this observational study.

## Results

Fifty-one deaths occurred in the PICU during the 12 months of the study. Forty children (78%) died following the decision to withdraw life-sustaining treatment while 5 (10%) died with some intensive care treatment being withheld. Six children (12%) died during resuscitation efforts. There was no case of confirmed brain death considered for organ donation. Complete datasets of 38 cases of withdrawal of life-sustaining treatment were recorded (75% of total PICU deaths, 95% of deaths following withdrawal of life-sustaining treatment). Three cases involved the withdrawal of extracorporeal life-support (1 ECMO, 2 LVAD) in addition to withdrawal of ventilation and inotropic infusions. Data of two cases were incomplete/unavailable. Distribution of age and diagnostic categories are displayed in table 1.

**Table 1:** Distribution of age and diagnostic categories amongst children in whom life-sustaining treatment was withdrawn

Parameter	Number	Percentage (%)
	n = 38	
**Age**		
Infant (0-12 months)	15	39
Young child (>1-4 years)	10	26.5
Older child (5-10 years)	5	13
Adolescent (11-17 years)	7	18.5
Young adult (>18 years)	1	3
**Diagnostic Category**		
Cardiac	11	29
Haematology/Oncology	6	16
Neurology	5	13
Hypoxic-ischaemic brain injury	4	10.5
Infectious Diseases	4	10.5
Respiratory	3	8
Gastrointestinal and metabolic	3	8
Trauma	2	5

**Figure 1:**
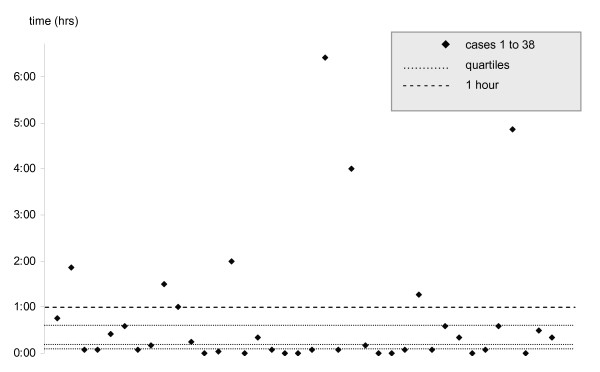
**Time from withdrawal of life-sustaining treatment to apparent death**.

The timeframes for addressing the issue of a withdrawal of life-sustaining treatment varied widely (Table 2). Similarly, significant time variations were recorded for the dying process. The median time from withdrawal of life-sustaining treatment to confirmation of death was 17 minutes (0.28 hrs.) ranging from immediate to a maximum of 6 hours and 28 minutes. Death was apparent at a median time of 10 minutes following withdrawal of life support varying from immediate to a maximum of 6.4 hours. The dying process took more than 1 hour in 8 of the 38 children (21%) (Figure 1). Medical confirmation of death took place from 0 minutes (minimum and median) to 35 minutes (maximum) thereafter with physicians having left the bedside during withdrawal in 18 cases (47%) to attend other patients or to give the family private time. The physician was present throughout the withdrawal process in 20 cases (53%).

**Table 2:** Timeframes of the decision-making process about withdrawal of life-sustaining treatment (hours)

Timeframe	Minimum	Median	Maximum
A - from first discussion between medical staff and parents to decision	0	7.75	457 (19d 1 hr)
B - from decision to withdrawal	0.5	4.71	47.33 (1d 23 hrs 20 min)
AB - from first discussion between medical staff and parents to withdrawal	0.75 (45 min)	27.21 (1d 3 hrs 13 min)	479.08 (19d 23 hrs 5 min)

In the vast majority of cases (35; 92%) all monitoring had been discontinued for withdrawal of life-sustaining treatment. Full monitoring (pulse oximetry, blood pressure, ECG, respiratory rate) had been continued in 2 cases and one case was managed with ongoing pulse oximetry only.

## Discussion

The proportion of cases of withdrawal of life-sustaining treatment among all unit deaths in our study is, at 78%, considerably larger than the percentages reported from other institutions. Studies from North America and the United Kingdom have described the percentage of withdrawal of treatment among the overall unit death rate between 60% and 65% [11,12] while authors from Europe and Brazil determined that proportion to lie below 50% [13,14]. The reasons for these differences may be diverse and include varying clinical practices, different attitudes, cultural backgrounds and, not least, changes in practice developing over time. Patient populations, too, may differ as some units, particularly in Europe, also practice neonatal intensive care [13]. Overall, however, the data of our study conform with the findings of other published research, in that the majority of deaths in the paediatric intensive care unit follows a decision to withdraw or withhold life-sustaining treatment rather than failed resuscitation efforts [15]. With regards to distribution of age and diagnostic categories, our data broadly reflect the patient population in an Australian PICU [16].

The nature of withdrawal of life-sustaining treatment prevents its study in randomised, controlled trials. Being observational and, as a study in a field with low mortality, inevitably being limited in the number of cases, our data must not be overinterpreted. The data is descriptive and merely depicts current clinical practice. Timeframes therefore should not be interpreted as benchmarks but instead observed as variables warranting examination and subject to a multitude of confounders, which are beyond control in this setting. The design of our study therefore strongly cautions against aiming to find correlations between the data. Its purpose was rather to facilitate understanding of a complex area of clinical practice by assembling a comprehensive picture of what until now has existed as fragmented pieces of data, records and subjective experience.

Nonetheless, having been conducted in a predominantly prospective, contemporaneous fashion, the study accurately describes current time-courses and clinical practice in a large, tertiary PICU. The data show extreme case-by-case variations in time at every step of the withdrawal process until death. Time-related information regarding the decision-making process is currently not available in the literature. Garros and colleagues, in a prospective survey, reported slightly less than half of end-of-life discussions requiring two or more meetings between the family and medical staff [12]. Our data describe the overall times from the first discussion between medical staff and parents about the subject to implementing the decision as a heavily skewed distribution. Most decisions are made and carried out within a day, yet only slightly less than half take longer and at times are drawn out considerably. Implementation of withdrawal of life-sustaining treatment appears not to be significantly postponed after the decision. In most cases this is done within less than 5 hours, however, a maximum delay of 2 days was also recorded. Taken as a whole, the intervals captured show the time-intensive and greatly variable nature of end-of-life discussions and decision-making in the PICU. In the context of our unit, decision-making is a shared process between medical staff and parents. It is, however, important to note that our study was not designed to record the time it may have taken within the PICU team to reach consensus before entering into dialogue with the parents.

With regards to the dying process, other reports had previously described approximate timeframes for the end of life based on retrospective studies. McCallum and colleagues recorded a timeframe from making a Do-Not-Resuscitate order to death of less than 24 hours [17] while Garros and colleagues determined a median time from decision to death of 3 hours [12]. In a more detailed audit, Zawistowski and colleagues described a timeframe of 30 minutes to 4.5 hours from withdrawal to death [18]. While being very informative in general, the confidence in the data obtained from these studies is limited by their retrospective nature, consequently having had to rely on the accuracy of patient records and narrative medical notes. We have endeavoured to capture the end of life contemporaneously and thus included not only the time of certification of death but also the time when death seemed apparent at the bedside in the absence of monitoring. Discontinuation of monitoring immediately before withdrawal of life-sustaining treatment is common practice in our unit in order to give parents time with their child with the least possible interference from medical technology. Confirmation of death, nevertheless, was not significantly delayed with most deaths having been certified immediately. A variety of reasons, however, may lead the physician to postpone this such as the wish to give the grieving family undisturbed time with the body of the child but also other urgent issues in the unit that need attending.

This notwithstanding, our prospective data confirm Zawistowski's finding that most children die within the first hour after withdrawal of life-sustaining treatment. Our data do, however, also show that this broad conclusion needs to be further qualified as some children die instantly following the withdrawal of treatment while, importantly, there also exists a great variation in the time to death. With a median time of 0.28 hours from withdrawal of treatment to confirmed death, the dying process occurred within less than 20 minutes in most children but took longer in just under half the cases. Most importantly, our data again provide evidence of considerable variations in timeframes from withdrawal of life-sustaining treatment to death. The relatively small number of cases and the variety of confounders in a non-controlled study setting prohibit correlating diagnosis or level of intensive care support with a time-course following withdrawal. Clinical acumen and intuition, on the other hand, have in the past been proven inaccurate in similar circumstances for more homogenous patient populations [19]. It is reasonable to assume that they, too, may not be reliable in predicting time to death.

For the doctor and nurse at the bedside, knowledge of this fact will be essential for planning the withdrawal process and the care for the child at the end of life. This will not only pertain to logistics and a pharmacological management plan but also to defining roles, duties and boundaries during what will be a process of unknown duration. Preparing the family for the dying process that is unpredictable in time may then help to guide parents and relatives during agonizing moments for which few will have points of reference. This may indeed offer an opportunity to prevent increasing anguish if the end of life is drawn out and give the family a better chance to cherish the last moments with their dying child.

Another layer of complexity is added to the end of life if organ donation after cardiac death (DCD) is considered. Current guidelines in Australia and New Zealand acknowledge the fact that time to death is unpredictable yet fail to address the practicalities of this circumstance [20]. In the context of DCD, the time to death assumes additional logistical importance to medical staff caring for the dying patient and may have added emotional significance to the grieving parents who have made the decision to donate the organs of their child if death occurs within a set timeframe. Limited, retrospective information about children considered for DCD in North America had to date yielded data of considerable disparity with regards to the time to death after withdrawal of life-sustaining treatment. Naim and colleagues, in a small series of 12 DCD candidates, found no child to have lived longer than 35 minutes after extubation [21]. Durall et. al. counted 14 out of 24 (58%) possible DCD candidates as having survived for more than one hour after withdrawal of life-support [22] while Pleacher and colleagues reported 2 out of 7 (29%) children who did not undergo planned DCD because of the dying process lasting more than 60 minutes [23]. Our prospectively collected data may offer encouraging information for proponents of DCD as the majority of deaths in the paediatric ICU did indeed occur within a narrow time span and 79% of children died within one hour, commonly given as the time limit within which organ procurement may occur. On the other hand, however, the unpredictability and great variation in times to death may make DCD impracticable in a large minority of cases (21% in our study). If organ procurement after withdrawal of life-sustaining treatment is contemplated, it is consequently essential that this unpredictability and variation in time to death is considered and addressed both when planning the process and in discussions with parents.

## Conclusions

There is a wide case-by-case variation in timeframes at every step of the process of withdrawal of life-sustaining treatment. Understanding the time-course of events in this important area of paediatric intensive care is essential for providing high-quality medical management, clinical leadership and guidance to parents at a most challenging time. The unpredictability and considerable variation in time to death may constitute a noteworthy challenge for accomplishing organ procurement after cardiac death.

## Abbreviations

DCD: Donation after Cardiac Death; ECMO: Extracorporeal Membrane Oxygenation; LVAD: Left Ventricular Assist Device; PICU: Paediatric Intensive Care Unit; RCH: The Royal Children's Hospital.

## Competing interests

JT declares that he has no competing interests. FO is affiliated with DonateLife Victoria.

## Authors' contributions

JT and FO jointly designed and conducted the study which had been conceived by JT. FO created the database, analysed as well as interpreted the data and drafted the article. JT and FO jointly revised the article. Both authors provided intellectual content of critical importance to this project and gave their final approval of this version to be published.

## Pre-publication history

The pre-publication history for this paper can be accessed here:

http://www.biomedcentral.com/1471-2431/11/39/prepub
